# The Seasonality Impact of the BTEX Pollution on the Atmosphere of Arad City, Romania

**DOI:** 10.3390/ijerph18094858

**Published:** 2021-05-02

**Authors:** Corina Popitanu, Gabriela Cioca, Lucian Copolovici, Dennis Iosif, Florentina-Daniela Munteanu, Dana Copolovici

**Affiliations:** 1 Biomedical Sciences Doctoral School, University of Oradea, 410087 Oradea, Romania; corinapascu@yahoo.com; 2 Preclinical Department, Faculty of Medicine, Lucian Blaga University of Sibiu, 550024 Sibiu, Romania; gabriela.cioca@ulbsibiu.ro; 3 Development and Innovation in Technical and Natural Sciences, Faculty of Food Engineering, Tourism and Environmental Protection, Institute for Research, Aurel Vlaicu University of Arad, 310330 Arad, Romania; dennisiosifnicu@gmail.com (D.I.); florentina.munteanu@uav.ro (F.-D.M.); dana.copolovici@uav.ro (D.C.)

**Keywords:** BTEX, air pollution, motor vehicles, health impact, urban air

## Abstract

Benzene, toluene, and total BTEX (benzene, toluene, ethylbenzene, and xylene) concentrations registered for one year (2016) have been determined every month for one high-density traffic area. The assessment was performed in Arad City, Romania, to evaluate these pollutants and their influence on the inhabitants’ health. The contaminants were sampled using a static sampling method and analyzed by gas chromatography coupled with mass spectrometry. Benzene was the most dominant among the BTEX compounds—the average concentrations ranged from 18.00 ± 1.32 µg m^−3^ in December to 2.47 ± 0.74 µg m^−3^ in August. The average toluene concentration over the year was 4.36 ± 2.42 µg m^−3^ (with a maximum of 9.60 ± 2.39 µg m^−3^ in November and a minimum of 1.04 ± 0.29 µg m^−3^ in May). The toluene/benzene ratio (T/B) was around 0.5, indicating substantial contributions from mobile sources (vehicles). The emission and accumulation of different aromatic compounds (especially benzene) could deteriorate the urban air quality. The lifetime cancer risk (LTCR) for benzene was found to be more than 10^−5^ in winter, including the inhabitants in the “probable cancer risk” category.

## 1. Introduction

Cities are more exposed to a higher concentration of pollutants because of the urban agglomeration of building, traffic, and industrial activities. As a result, the concentration of different pollutants tends to be a hundred times higher in cities than in rural areas. Such an increase in carbon source emissions determines haze formation and decreases the ecological and environmental conditions [1]. As a consequence, the health of the inhabitants of the cities is affected. Recently, the synergic effect of heat (which is due to climate change) and air pollution (as particulate matter and different organic compounds) on human health has been shown [2].

In cities, the emission of BTEX (benzene, toluene, ethylbenzene, and xylene) compounds becomes higher, mainly due to intensive industrialization and urbanization. BTEX are emitted from both anthropogenic and natural sources and are implicated in forming ozone and photochemical reactions in the atmosphere [3]. Benzene and other aromatic compounds are important precursors of secondary organic aerosols formed by photochemical oxidation reactions and heterogeneous reactions in the presence of solar radiation and nitrogen oxides (which are usually present in the pollutant atmosphere) [4,5]. Numerous studies measured major pollutants within and outside urban areas of different cities worldwide [6,7,8,9,10,11].

In Europe, 40 years of investigations have revealed that due to the implementation of EURO (European emission standards) standards for vehicles (for NOx, CO, volatile organic compounds), transport emissions have been reduced, contributing to a positive impact on air quality in Europe [12]. The chemical composition of air pollutants emerging from Europe has been determined in the Cyprus Photochemistry Experiment, which revealed high VOC concentrations across the Mediterranean Sea [13,14]. The photochemical reactions in the atmosphere of aromatic compounds (benzene, toluene, xylene) have been studied using an outdoor simulation chamber, such as the EUPHORE smog chamber [15,16] and the Simulation of Atmospheric Photochemistry In a large Reaction Chamber (SAPHIR) [17]. These studies are well correlated with the measurements performed in urban or forest atmospheres. The results obtained in these chambers have been used to explain the chemical reactions in the atmosphere [18].

For studying the urban ambient air, different approaches can be used. It is possible to carry out long-term period sampling strategy studies regarding the determination of BTEX in the urban atmosphere. For example, there are differences found in ambient air in industrial, residential, or commercial areas around Yokohama city, Japan, between summer and winter, with the highest concentrations of BTEX in industrial areas [19]. A study during two climatic seasons of 2018 (summer and autumn) for BTEX determination has been conducted in Leon, Guanajuato, Mexico, which showed high–medium concentrations of toluene, ethylbenzene, and p-xylene during summer and the highest concentration of benzene in autumn [20]. In Jeddah, a coastal city in Saudi Arabia, the BTEX determinations showed seasonal variation during a one-year experiment, with higher concentrations during the spring and lower concentrations during the autumn [21]. The results obtained after a long-term (2011–2013) determination of volatile organic compounds in Moskow, Russia, revealed that BTEX made a lesser contribution to O_3_ formation in the city [22].

However, measurements could be performed only for a short time, e.g., considering only one season. The seasonality of volatile organic compounds and different pollutant concentrations becomes more critical as, in some months, these compounds affect human health more than in others [23,24]. In a study regarding the BTEX pollution in Delhi, India by Garg et al. [25] it was shown that in a high-traffic intersection area, large differences between values were recorded in winter and summer (86.84 ± 32.55 μg m^−3^ and 68.35 ± 48.26 μg m^−3^, respectively). In other work, it has been demonstrated that such a large difference between winter and summer pollution data could be due to local sources, such as industry and transportation [26]. The concentration changes in the emission of pollutants in different seasons have been observed in both urban and remote sites [27,28]. The emission of BTEX compounds in Salvador (Brazil) was reported at a level of 5.90 ± 3.28 µg m^−3^ in the dry period and 7.95 ± 2.95 µg m^−3^ in the wet period [29].

Regarding the data related to volatile organic compound emission in Romania, there are only two articles that have been published detailing the total volatile organic compounds over a medium Romanian city—Mures County—with 32,000 inhabitants (176.72–192.54 μg m^−3^) [30] and over the Olt River Basin, the principal affluent of the Danube River [31]. The number of cars in Romania is continuously increasing, from 1.54 million in 1990 to 5.92 million in 2014 [32], while the Romanian Statistics Institute reported 8.7 million on 31st of December 2019. It should be noted that around 79% of these cars are at least ten years old. Therefore, the primary source of pollution in the cities is traffic, contributing to air pollution by more than 25% [33].

The occurrence of pollutants affects not only the ambient air quality but also increases human respiratory symptoms and deaths. The scientific literature shows that respiratory problems, acute bronchitis, heart problems, lung cancer, lung diseases, and asthma have increased due to the high pollution levels of BTEX (especially benzene, which is classified as “carcinogenic to humans” (Group 1)) in the environment [20]. A recent meta-analysis study has shown morbidity in some months for cardiovascular and respiratory diseases, mainly for stroke and pneumonia. The effect is even more pronounced for children and the elderly [34].

In this study, we performed the determination of BTEX levels during one year in a high-density traffic area from Arad, Romania, to evaluate the magnitude of seasonal variability of BTEX concentrations. For the first time, we then correlated these data with the potential impacts of these pollutants on public health in Romania.

## 2. Materials and Methods

### 2.1. Field Measurement Site

**Figure 1:**
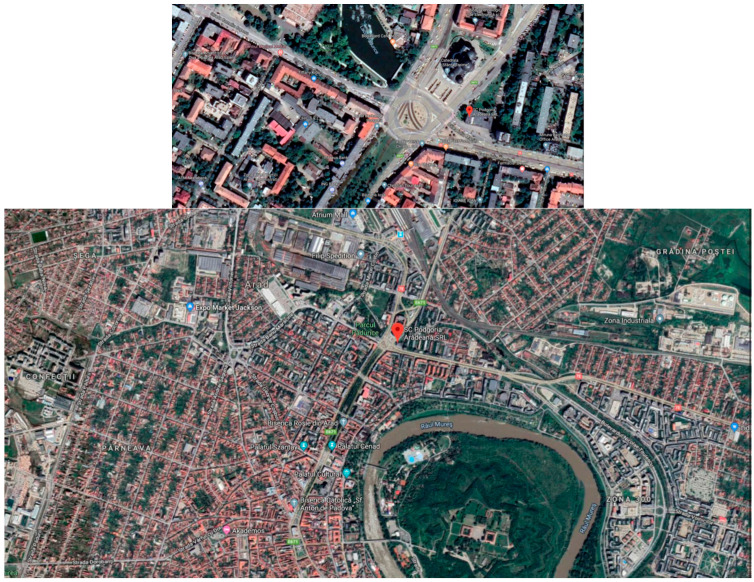
Satellite photos of Arad City Center. The position of the sampling site is marked with a red pin.

In this study, the chosen sampling site is a high-density traffic area (Podgoria, the main crossroad in the town) in Arad City (173,000 inhabitants), in the western part of Romania (coordinates: latitude: 46°10′59.99″ N, longitude: 21°19′0.01″ E) (Figure 1).

### 2.2. Sampling and Analysis

The present study determined BTEX concentrations (benzene, toluene, ethylbenzene, and xylene) in different seasons—winter, spring, summer, and autumn—in the main crossroad from Arad City, from January to December 2016. Every month, there were four sampling times (every time, four samples were collected simultaneously—in total, 16 samples). Sampling was carried out at the height of 2 m above ground level by using air pumps (SKC 1003, SKC Inc., Houston, TX, USA), and stainless steel tubes (10.5 cm length, 4 mm inner diameter, Supelco, Bellefonte, PA, USA) filled with a mixture 2:1:1 *w/w/w*, Carbotrap C: Carbopack C: Carbotrap X adsorbents (Supelco, Bellefonte, PA, USA), with a flow rate of 200 mL min^−1^ for 60 min. Before use, tubes were conditioned for 30 min at 350 °C in a pure He flow of 50 mL min^−1^. Four samples were collected every time, and the tubes were analyzed on the same day as the sampling. The separation and detection of the compounds were performed by GC–MS (Shimadzu 2010 plus, GCMSTQ8040, Tokyo, Japan) coupled with a thermal desorption system (Shimadzu TD20, Kyoto, Japan) as described in Kannaste et al. [35].

To establish correlations between BTEX and the formation of tropospheric ozone in different seasons, the ozone concentration data were taken from open access data available at http://www.anpm.ro (accessed date: 17 June 2017).

### 2.3. Statistical Analysis

GraphPad Prism software (version 5.0 for Windows, GraphPad Software, San Diego, CA, USA) was used for statistical analysis. An analysis of variance (ANOVA) was undertaken for the identification of statistically significant comparisons and the least significant difference (LSD) calculations at an alpha level of 0.05 (α = 0.05). Spearman’s correlation was applied for the identification of the correlation coefficient among BTEX species and ozone.

### 2.4. The Health Risk Assessment

The US EPA recommends the inhalation route method for the health risk assessment (HRA) of BTEX. The procedure is described in detail in Latif et al. [36]. The chronic daily intake (CDI) (mg m^−3^) was calculated using Equation (1):

CDI = (CA × CF × IR × ET × EF × ED)/(BW × AT)(1)

where

CA = contaminant concentration in air (mg m^−3^)
CF = conversion factor (0.001 mg mg^−1^)
IR = inhalation rate (m^3^ h^−1^) for an adult (0.83)
ET = exposure time (24 h day^−1^)
EF = exposure frequency (350 days year^−1^)
ED = exposure duration (24 years for an adult)
BW = body weight (70 kg for adults)
AT = averaging time (ED in years × 365 days year^−1^) for noncarcinogenic risk calculation
AT = ¼ averaging time (70 years × 365 day year^−1^) for carcinogenic risk calculation

Hazard quotient (HQ) for toluene was estimated as in Equation (2):

HQ = CDI/RfC(2)

Inhalation reference concentration (RfC) = 5 mg m^−3^.

The lifetime cancer risk (LTCR) was calculated following the US EPA method (as described in [36]). Equation (3) was used to calculate the LTRC for benzene:

LTCR = CDI × SF(3)

where SF (carcinogenic slope factor) = 0.0273 (mg (kg-day)^−1^)^−1^.

## 3. Results

**Figure 2:**
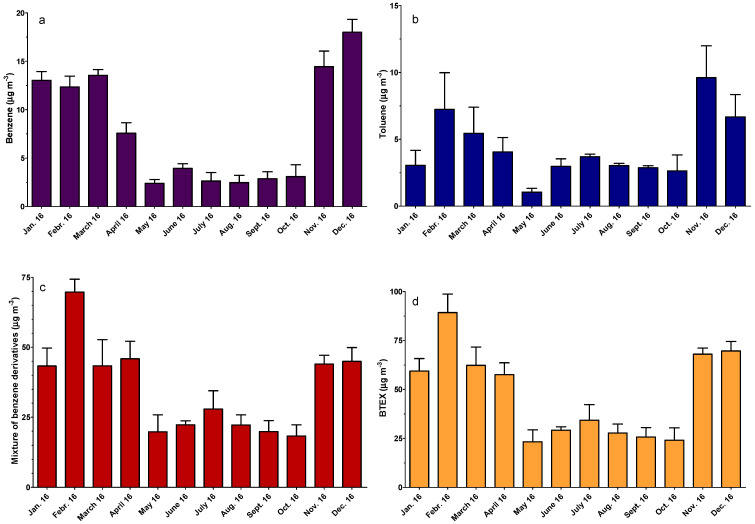
Monthly variations in benzene (**a**), toluene (**b**), benzene derivatives (o-, p-xylene, and ethylbenzene) (**c**), and total BTEX (**d**).

Figure 2 illustrates the variability of BTEX concentrations over 12 months, monitored in a high-traffic crossroad in Arad City, Romania. The results of the one-way ANOVA show that there is a statistically significant difference during the year for all these compounds. In the case of benzene, the concentrations were not statistically different in the cold months (until April), while in the hot season, there was a drop in these values. The highest BTEX levels were found in winter (February), when a mean value of 89.29 μg m^−3^ was registered, and the lowest levels of BTEX compounds were measured in autumn (October), with a determined concentration of 24.01 μg m^−3^.

The variability observed during the 12-month monitoring period is shown in Figure 2.

The toluene concentration was higher in winter (December–February, with a maximum of 9.60 μg m^−3^ in December) compared with the spring and summer months (with a minimum of 1.04 μg m^−3^ in May). The registered values for the mixture of benzene derivatives (o-, p-xylene, and ethylbenzene) were lower, especially in the summer months.

An indicator of traffic emissions could be assessed by calculating the toluene/benzene (T/B) ratios. In the present study, the T/B ratios were less than one for most months, with the lowest value found in January. In contrast, the T/B ratio became more than one in the hot summer months (Table 1).

**Figure 3:**
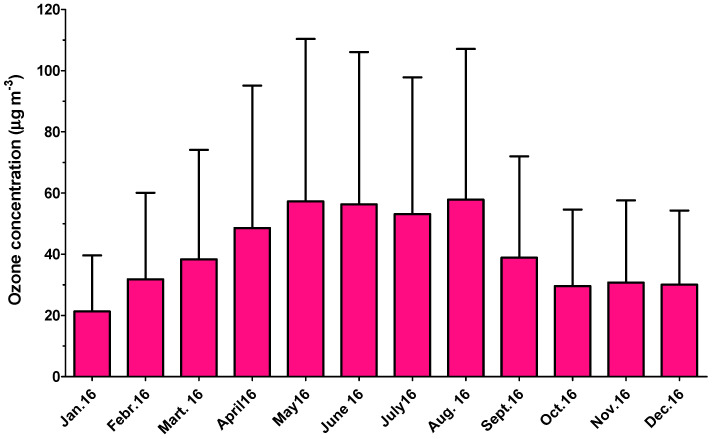
Monthly variation of ozone concentration.

The trend of ozone concentration over the year is presented in Figure 3. The medium concentration did not exceed 75 μg m^−3^, even in summer. However, the maximum concentration of ozone often exceeded 100 μg m^−3^.

The non-parametric correlation between values of pollutant concentrations reveals strong correlations between all BTEX. In contrast, a weak correlation was found between toluene and benzene derivatives and ozone (Table 2).

In order to estimate the health effects for inhabitants exposed to BTEX, the chronic daily intake (CDI) values for all compounds were evaluated (Table 3).

For benzene, the mean CDI value of 0.75 μg day^−1^ kg^−1^ in the crossroad was almost three times higher than that of the city’s mean value (0.26 μg day^−1^ kg^−1^). Toluene had CDI values that vary from 0.89 μg day^−1^ kg^−1^ in November and 0.09 μg day^−1^ kg^−1^ in May, while total BTEX ranged from 8.35 μg day^−1^ kg^−1^ to 2.16 μg day^−1^ kg^−1^ with a mean value of 4.44 μg day^−1^ kg^−1^.

## 4. Discussion

As the measurements were taken in the middle of an intersection with heavy traffic, the annual mean values for benzene were much higher than the legal limits; according to Directive 2000/69/E.C., the annual mean values must not exceed 5 μg m^−3^. Indeed, the annually measured concentration for benzene over the city in the daytime was 2.87 ± 0.58 μg m^−3^. The average concentration of benzene for different cities is presented in Table 4. The emission level in Arad was similar to that in Teheran but higher than in other cities.

Our study shows high mean values for benzene (2.87 ± 0.58 µg m^−3^) that are comparable with the mean values registered for a hospital garage (the 24-h mean concentration of 6.78 μg m^−3^) [43].

For toluene, the level (mean value of 4.36 ± 2.42 μg m^−3^) remained below the threshold established by the World Health Organization (WHO) at 260 μg m^−3^ as a weekly average for protecting human health (Figure 2b). The same concentration level was found in different studies related to measurements near roads [36,44,45]. The total BTEX concentration varied between 23.17 μg m^−3^ and 89.29 μg m^−3^, which is lower than the total BTEX measured at the roadside in Malaysia (131 μg m^−3^) [46], in China at the northern part of the First Ring Road of Hefei, in a rural tunnel in Bilecik, Turkey [47], or at near-road schools in El Paso, TX, USA [48] (Figure 2d).

Urban ambient BTEX emission sources are the primary outcome of human activities. The ratio among aromatic compounds is a significant indicator in studying the pollution source. In our case, the ratio between toluene and benzene (T/B) was less than one (Table 1), which is indicative of substantial contributions from mobile sources (vehicles) [49]. As benzene was emitted by vehicular sources, and toluene was released from both mobile and point sources, the industrial emission was marginal in our case. Indeed, the T/B ratio in Arad City was 0.54, which is an indicator of substantial contributions from mobile sources. A low T/B ratio was reported for sampling points with traffic as the primary emission source [50,51]. The reaction of benzene and toluene with hydroxyl (OH) radicals is the primary factor that leads to a decrease in toluene and benzene concentrations. Therefore, significantly lower ambient T/B ratios show that the emission from mobile sources came from a distance and is expected to have traveled and degraded. In contrast, higher T/B ratios may reflect relatively fresh vehicular emission sources. As the reaction rate constant of the toluene reaction with hydroxyl (OH) radicals was approximately five times higher than that for benzene [52], the sources of toluene emission were far from the city center, and mass transportation was low.

Such a pattern could be related to two facts: firstly, traffic is lower in the summer than in the winter, as many inhabitants leave town for holidays, and secondly, the city’s central heating sources are not in use. In addition, the ozone concentrations (Figure 3) are much higher in the summer months than in cold months, and as a consequence, the atmospheric oxidation of benzene can occur. In contrast, the toluene concentrations do not drop dramatically in summer, as industry is the primary emission source. In any case, there is a slight decrease in its concentration in summer, which could be related to its reaction with ozone and OH radicals during long transport, and also to a small decrease due to the holidays and a possible reduction in industrial activity.

The patterns of total BTEX concentration, which increases only in November and start to decrease in March, can be explained by the fact that in Arad City, central heating starts to be used at the beginning of November. The registered medium temperature in October, in Arad, over more than 100 years (1880–2018), is +17 °C, while in February, the medium temperature is +4 °C. As found in many other studies, BTEX concentrations were generally higher in winter and autumn than in spring and summer [26,28,50]. BTEX are removed faster in summer than in winter due to the higher reaction rates with OH radicals in the atmosphere [52]. The high concentration in winter is due to higher atmospheric stability, while in summer, atmospheric dispersion becomes essential. The formation of a tropospheric ozone in Arad, Romania in the summertime is due to the high photochemical activity involved in the contribution of BTEX. Similar results have been found in Monterrey, Mexico [53].

The strong non-parametric Spearman’s correlations between toluene and benzene derivatives (xylenes and ethylbenzene) and between toluene and total BTEX indicate that the emission sources of these compounds may be similar. The same results have been shown in other studies in which there are robust correlations between aromatic pollutants [54,55]. The lower correlation between BTEX and ozone could be because, in the photochemical reactions, more volatile organic compounds than BTEX are involved in the tropospheric ozone formation [56].

The chronic daily intake (CDI) values (Table 3) are in the same range as those determined in different Malaysian sites [36]. The HQ values for toluene over the year are far below 1, indicating no significant risk to human health relating to this compound. Benzene is classified as carcinogenic to humans (Group 1) by the International Agency for Research on Cancer (IARC), and its maximum concentration is established as 5 µg m^−3^. In our case, the estimated LTCR for benzene revealed values over 3 × 10^−5^ in winter, which is in the “probable cancer risk” category, while in summer, the values were less than 10^−5^, which indicates a “possible cancer risk”.

Considering the influences of BTEX on human health, national and local policymakers should implement international regulations and increase both accessibility to the new-energy vehicle industry products [57] and the ecological behavior of the social capital [58].

## 5. Conclusions

A one-year dataset of atmospheric concentrations of BTEX in the busiest crossroad in Arad City, Romania, was compiled in order to observe the implications for human health. The temporal variability of total BTEX concentration showed a higher concentration in winter than summer and low chronic daily intake (CDI). The lifetime cancer risk (LTCR) for benzene was in the “probable cancer risk” category in winter, while in summer, the risks were minimal for humans. The present study underlines the importance of BTEX determination in cities with industrial activities and heavy traffic—especially in Eastern Europe, where many old vehicles are used. More studies are necessary to identify and control the emission sources of pollutants to indicate a possible recommendation to increase the quality of the urban atmosphere, such as using new, electric vehicles, upgrading the public transport system, and regulating emissions (from industry, heating systems, and transportation).

## Figures and Tables

**Table 1:** The T/B ratios over the year in the ambient atmosphere in Arad City.

Month	Jan.	Feb.	Mart.	April	May	June	July	Aug.	Sept.	Oct.	Nov.	Dec.
T/B ratio	0.23	0.59	0.40	0.54	0.43	0.76	1.41	1.22	1.00	0.85	0.66	0.37

**Table 2:** Spearman’s correlation coefficients, r, for the studied compounds.

Pollutant	Benzene	Toluene	Benzene Derivatives	BTEX	Ozone
Benzene	1.000				
Toluene	0.776	1.000			
Benzene derivatives	0.720	0.909	1.000		
BTEX	0.888	0.951	0.923	1.000	
Ozone	0.713	0.488	0.287	0.476	1.000

**Table 3:** Chronic daily intake (CDI), hazard quotient (HQ), and lifetime cancer risk (LTCR) of BTEX determined in Arad City.

Month	CDI (µg day^−1^ kg^−1^)				HQ	LTCR
	Benzene	Toluene	Benzene Derivate	BTEX	Toluene	Benzene
Ian.	1.22	0.28	4.05	5.56	2.03 × 10^−4^	3.33 × 10^−5^
Feb.	1.16	0.68	6.52	8.35	4.83 × 10^−4^	3.15 × 10^−5^
Mart.	1.27	0.51	4.05	5.83	3.63 × 10^−4^	3.46 × 10^−5^
April	0.71	0.38	4.29	5.38	2.71 × 10^−4^	1.93 × 10^−5^
May	0.22	0.10	1.85	2.17	6.97 × 10^−5^	6.13 × 10^−6^
June	0.37	0.28	2.08	2.73	1.99 × 10^−4^	1.00 × 10^−5^
July	0.25	0.35	2.61	3.20	2.47 × 10^−4^	6.72 × 10^−6^
Aug.	0.23	0.28	2.08	2.59	2.02 × 10^−4^	6.32 × 10^−6^
Sept.	0.27	0.27	1.86	2.40	1.92 × 10^−4^	7.33 × 10^−6^
Oct.	0.29	0.25	1.71	2.25	1.76 × 10^−4^	7.89 × 10^−6^
Nov.	1.35	0.90	4.11	6.36	6.42 × 10^−4^	3.69 × 10^−5^
Dec.	1.68	0.62	4.21	6.51	4.46 × 10^−4^	4.60 × 10^−5^

**Table 4:** The average concentration of benzene in different cities.

City	Benzene Concentration (µg/m^3^)	Reference
Arad, Romania	2.87 ± 0.58	Present study
Berlin, Germany	0.82 ± 0.45	[37]
Budapest, Hungary	0.89 ± 0.67	[37]
Mons, France	0.57 ± 0.45	[37]
Torino, Italy	0.63 ± 0.57	[37]
Gdansk, Poland	0.75 ± 0.67	[38]
Gdynia, Poland	0.66 ± 0.51	[38]
Sopot, Hungary	0.63 ± 0.55	[38]
Nuevo Leon, Mexico	0.65	[39]
Gorakhpur, India	12.1	[40]
Delhi, India	8.98 ± 4.72	[41]
Teheran, Iran	2.57	[42]
